# Video WeAther RecoGnition (VARG): An Intensity-Labeled Video Weather Recognition Dataset

**DOI:** 10.3390/jimaging10110281

**Published:** 2024-11-05

**Authors:** Himanshu Gupta, Oleksandr Kotlyar, Henrik Andreasson, Achim J. Lilienthal

**Affiliations:** 1 Centre for Applied Autonomous Sensor Systems, Örebro University, 701 82 Örebro, Sweden; 2 Perception for Intelligent Systems, Technical University of Munich, 80333 München, Germany

**Keywords:** weather detection, video classification, weather intensity classification

## Abstract

Adverse weather (rain, snow, and fog) can negatively impact computer vision tasks by introducing noise in sensor data; therefore, it is essential to recognize weather conditions for building safe and robust autonomous systems in the agricultural and autonomous driving/drone sectors. The performance degradation in computer vision tasks due to adverse weather depends on the type of weather and the intensity, which influences the amount of noise in sensor data. However, existing weather recognition datasets often lack intensity labels, limiting their effectiveness. To address this limitation, we present VARG, a novel video-based weather recognition dataset with weather intensity labels. The dataset comprises a diverse set of short video sequences collected from various social media platforms and videos recorded by the authors, processed into usable clips, and categorized into three major weather categories, rain, fog, and snow, with three intensity classes: absent/no, moderate, and high. The dataset contains 6742 annotated clips from 1079 videos, with the training set containing 5159 clips and the test set containing 1583 clips. Two sets of annotations are provided for training, the first set to train the models as a multi-label weather intensity classifier and the second set to train the models as a multi-class classifier for three weather scenarios. This paper describes the dataset characteristics and presents an evaluation study using several deep learning-based video recognition approaches for weather intensity prediction.

## 1. Introduction

Knowing weather conditions and their intensity is critical for reliable scene awareness in autonomous navigation, autonomous systems in the agriculture sector, and emerging technologies like quadcopters/drones. Adverse weather can negatively affect the sensors—cameras, lidars, and radars—typically used in these applications. The camera sensor is a passive sensor that depends on ambient light, which varies depending on the time of the day and weather; in cases of adverse weather like snow, fog, and rain, the visibility of objects in the camera image is reduced due to low illumination and added noise. Lidar sensors are not affected by ambient light but project fog, rain drops, and snowflakes as points representing noise. Adverse weather also affects radar data but not to the extent of lidars and camera sensors [1]. Tackling the noise issue due to adverse weather or determining the reliability of the information from sensors in data post-processing requires knowledge about the current weather. However, knowing the current type of weather is insufficient, as the amount of noise in the sensor data is also directly related to the intensity of adverse weather; hence, knowledge of intensity is quite valuable.

While weather intensity information is available through weather prediction technology, it is typically estimated for larger regions and may not accurately reflect local conditions. Moreover, weather predictions only sometimes align with the actual conditions and instantaneous changes (in the case of underground mines or mountain regions), leading to potentially incorrect information. Consequently, a camera-based approach is more suitable for weather recognition and intensity estimation, considering cameras are often part of the sensor suite in autonomous navigation systems.

Weather recognition has been part of computer vision research for quite some time for scene understanding, especially for on-road conditions. Earlier work focused on using analytical approaches for detecting single weather labels like detecting rain intensity as proposed in [2] or detecting fog on highways as proposed in [3]. In contrast, recent works have focused on deep learning-based approaches for multi-label weather classification using camera images. In [4], a new multi-label weather dataset was published with six weather labels (sunny, cloudy, fog, snow, moist, and rain) along with a deep neural network. However, no information on the weather intensity was provided. Furthermore, the “snow” label does not represent the snowing condition in this dataset. In [5], a new network called WeatherNet was proposed for detecting weather (snow, rain, clear), time of day (day, night, dusk/dawn), fog, and camera glare using a combination of multiple ResNet50 networks. However, the dataset created and used in [5] does not have an intensity label and is not publicly available. Apart from these, several other image-based datasets are available for detecting single weather labels like [6] for fog detection and [7] for rain denoising containing rain/rain-free image pairs. However, none of these datasets contain information about weather intensity. A recent image dataset, WeatherKitti, with a weather intensity label was proposed in [8] for the purpose of monocular depth estimation in adverse weather conditions. However, the adverse weather is synthetically generated using Generative Adversarial Networks and may not reflect real weather conditions.

To address this gap, we introduce the Video WeAther RecoGnition (VARG)dataset—an innovative and challenging dataset with two sets of weather intensity labels (for multi-label and multi-class classification) designed explicitly for weather intensity detection using deep learning-based video recognition methods. We trained multiple video-based deep learning models, including 2D CNNs, 3D CNNs, and transformer-based models on the VARG dataset for weather intensity prediction, and we present a comprehensive evaluation that demonstrates the effectiveness of weather intensity prediction using video clips. This weather and intensity information is crucial for making informed decisions about sensor reliability, for prediction score thresholds for object detection tasks, and for determining the filtering algorithms for removing noise from lidar sensor data in adverse weather conditions, as shown in Appendix A.

## 2. Dataset

**Figure 1:**
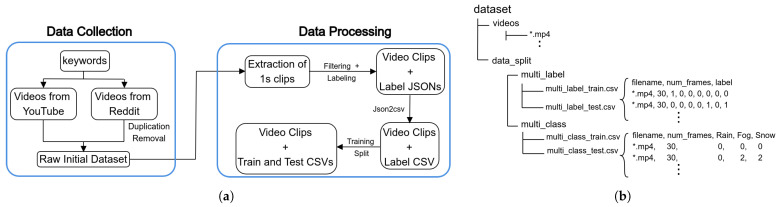
Illustration of dataset construction pipeline and the dataset directory structure. (**a**) Dataset construction pipeline. (**b**) Dataset folder structure.

The VARG dataset was created to provide a valuable resource for weather intensity prediction in adverse weather conditions. It was constructed by collecting videos of adverse weather from various social media platforms, supplemented by original clips captured by the authors. The collected videos were processed into 1-s clips, comprising approximately 25–30 frames, and manually annotated. This section elaborates on the data collection process, the rationale behind manual annotation, and the specific labeling guidelines applied for each weather category. The dataset construction pipeline and the dataset structure are shown in Figure 1.

### 2.1. Dataset Collection

While several real-world autonomous driving datasets exist, incorporating adverse weather scenarios (like the Radiate dataset [9], Boreas dataset [10], CADC [11] etc.), they are not ideal candidates for the creation of a weather intensity prediction dataset due to various limitations. For example, these datasets typically lack weather timestamps and weather intensity information and have an imbalanced distribution biased towards clear weather and low weather intensity. Consequently, processing and annotating such datasets would yield limited benefits regarding weather intensity prediction. Hence, a more comprehensive dataset was created to address these shortcomings, encompassing diverse scenes, camera dynamics, time of day, and a more balanced representation of different weather types and intensities.

Numerous videos depicting adverse weather were sourced from YouTube and Reddit. The collection process involved a semi-automated search-and-download approach. For YouTube videos, a combination of keywords such as “snowing”, “fog”, “snow”, “rain”, “dashcam”, “walk-through”, “city”, and “forest” was employed to locate relevant content. The Pytube library (version 12.1.3) facilitated the download of selected videos. In the case of Reddit, videos from specific Subreddits such as “rain”, “raining”, “snow”, “fog”, and “dashcam” were collected using the reddit-crawler library (https://github.com/Kumar-Kishan/reddit-crawler, accessed on 25 November 2021).

**Figure 2:**
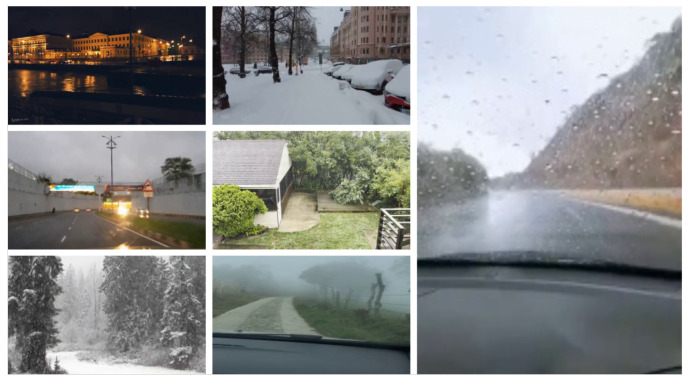
Examples demonstrating the variety in the VARG dataset regarding scenario, camera dynamicity, time of day, and image quality.

Overall, the dataset consists of 110 videos sourced from YouTube, 939 videos from Reddit, and 30 videos captured by the authors, providing a diverse range of adverse weather scenarios for a well-balanced weather intensity recognition dataset. The collected videos exhibit variations in scene types (urban, suburban, rural, forest, and parks), camera dynamics (static cameras, hand-held cameras, dashcams, and drones), time of day (daytime, dusk/dawn, and night), as well as frame size and quality, as illustrated in Figure 2.

### 2.2. Dataset Preparation

The videos collected from YouTube and Reddit have varying lengths, with not all frames containing discernible weather-related details. Weather intensity often fluctuates within a single video, making it unfeasible to assign a single label to the entire video. Moreover, a video file might encompass different weather intensities due to a compilation video of smaller clips. The collected video data were processed to extract usable clips to ensure data consistency.

Multiple 1 s clips, comprising approximately 25–30 frames, were extracted from each video during the data preparation phase. The number of clips extracted depended on the length of the original video. The clips were split into overlapping upper and lower regions for portrait mode but left unchanged for landscape mode. Only a few extracted clips contained pertinent information for weather recognition. Hence, each clip was labeled for usability during the data labeling process. The criterion for clip usability was based on the presence of visible weather-related information in each frame. It is worth noting that the videos within the dataset maintain considerable variation in terms of frame size and aspect ratio, intentionally left unstandardized during the dataset preparation stage and left for the dataset users to decide upon.

### 2.3. Dataset Labeling

We annotated the collected clips to have three weather types, rain, fog, and snow, each with three intensity classes: absent/no, moderate, and high. The “absent/no” intensity label corresponds to the absence of that weather phenomenon. The weather intensity is divided into two qualitative labels (moderate and high) because downstream computer vision tasks like object detection, segmentation, and scan registration using a camera or a lidar sensor are severely affected in heavy weather intensity conditions and fare well in moderate weather conditions with some noise in sensor data [12,13]. Hence, we considered only two qualitative labels for weather intensity classification.

#### 2.3.1. Auto-Annotation of Weather Intensity?

**Figure 3:**
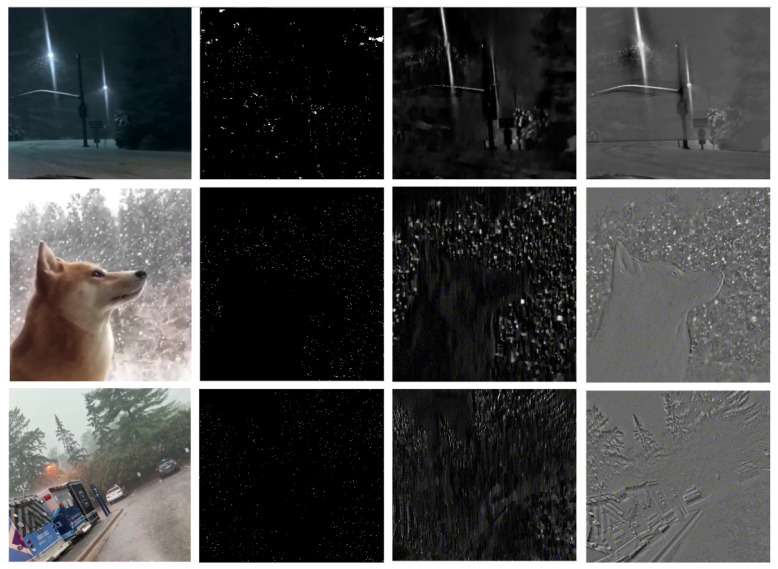
Rain streak and snowflakes detection using [14] (2nd column), [15] (3rd column), and [16] (4th column). Top row: dynamic background, heavy snowfall, and low illumination. Middle row: state background, heavy snowfall, and well illuminated. Bottom row: dynamic background, heavy rainfall, and well illuminated.

An auto-annotation approach for intensity labeling was explored by detecting the rain streaks and snowflakes in the videos using analytical denoising methods. Analytical methods were selected instead of deep learning approaches due to the diversity of datasets, which poses a significant challenge to current deep learning denoising methods. Three different methods for rain denoising were investigated. The first method involved modeling the physics of rain phenomena, as proposed in [14]. The second method, FastDerain [15], utilized directional gradient priors. The third method relied on tensor decomposition to obtain a low-rank sparse matrix that models moving objects [16]. The reimplementation of the [14] method by [17] was used, alongside the implementation of FastDerain provided in the original paper [15]. The LRSLibrary [16] was employed for calculating the low-rank sparse matrix. However, these methods relied on the assumption of a static background, which did not hold for a significant portion of the dataset. Additionally, factors such as illumination source, falling direction, and noise size further complicate rain streak and snowflake detection in real-world scenarios. Figure 3 illustrates the results of rain streak and snowflake detection using the methods above on some video clips.

#### 2.3.2. Manual Labeling

Due to the absence of a direct means to precisely measure weather intensity from images or videos and the limitations of the auto-annotation approach, manual annotation was employed for weather intensity labeling. Given the complexity of weather phenomena, the annotations were based on a visibility-based classification of weather, taking into account its impact on sensor data noise. The Label Studio open-source library [18] was utilized for the manual annotation of the dataset.

#### Fog

The visibility-based classification proposed in [19] for use in individual road traffic conditions was used as a reference to label fog intensity. According to [19], the fog intensity is low if visibility is between 0.3 and 1 km, moderate if visibility is between 100 and 300 m, and dense/high if visibility is less than 100 m. However, an exception was made to categorize distant fog as clear, as it does not impact camera or lidar sensor visibility for autonomous driving tasks as the usable ranges for lidar and radar sensors are 100–150 m and 300 m, respectively.

#### Snow

The American Meteorological Society’s visibility-based classification for snow weather observation was a reference for snow intensity labeling. Snow intensity was classified as low when visibility was at least 1 km, moderate for visibility between 0.5 and 1 km, and high for visibility less than 0.5 km. The low- and moderate-intensity classes were merged into a single category. Snow intensity labeling also considered factors such as snowflake size and snowfall rate as these affect the noise in the camera and lidar sensor’s data streams, which are considered while labeling.

#### Rain

The rain intensity is measured as the amount of rainfall (rainfall depth) in a specific time interval, and no visibility-based classification is available. However, heavy rain has a similar effect on visibility as moderate fog, which helps distinguish the rain intensity from mild or moderate. Moreover, similar to snow labeling, the rainfall rate and amount of rain streaks were considered during rain intensity labeling.

As the weather intensities annotation was performed manually, it has the personal bias of the annotator. To reduce personal bias’s impact on annotations and provide consistent annotations for weather intensities for the dataset, the labeling was performed three times for the whole dataset with a two-week gap. In addition, the weather intensity labeling was performed using the full videos as a reference.

The VARG dataset contains only a minimal number of clips with identifiable faces, and none are prominently featured. The dataset was curated from publicly available and self-collected videos and did not include any sensitive personal information (https://commission.europa.eu/law/law-topic/data-protection/reform/rules-business-and-organisations/legal-grounds-processing-data/sensitive-data/what-personal-data-considered-sensitive_en (accessed on 6 October 2024)), such as political opinions, biometric data, or health-related information.

#### 2.3.3. Dataset Labels

**Figure 4:**
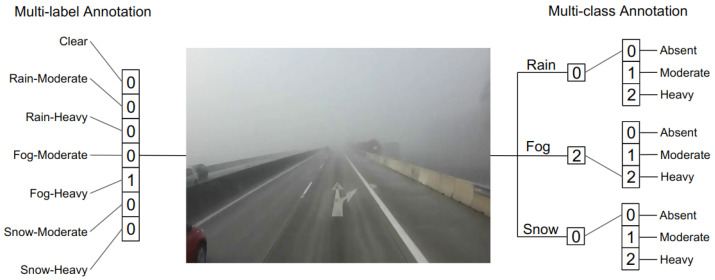
Examples of the two sets of annotation provided in VARG dataset.

The annotated video labels are processed into two sets of annotations. The first annotation set has seven labels (clear, rain–moderate, rain–heavy, fog–moderate, fog–heavy, snow–moderate, and snow–heavy) and could be used to train a multi-label video classifier. The second set provides labels for multi-class intensities classification for three weather types that can be used to train a video classifier with three independent classifiers for three weather types, rain, fog, and snow. In the multi-class annotation set, the clear weather corresponds to the “absence” of all three weather types. Figure 4 illustrates the two sets of labels provided with the dataset for a given clip. We provided two annotation sets to make the dataset more generalized and give the dataset user more choice. The multi-class annotations can be used to enforce exclusivity between weather-intensity classes, and the multi-label annotation set can be used to make the weather-intensity labels more inclusive.

### 2.4. Dataset Details

The number of clips for each weather label in the VARG dataset based on weather intensity is given in Table 1. The dataset has clips with multiple kinds of weather that co-occur, specifically rain–fog and snow–fog. For example, the number of clips with rain–fog labels is 49 and with snow–fog labels is 1495. However, our dataset lacks clips with cases of rain–snow and rain–snow–fog.

The dataset was split into train and test sets to have an 80/20 proportion of clips for each weather label and an even distribution of the weather and intensity labels. The clips in the test set and the train set are taken from different videos and, hence, do not overlap. In addition, the clips in the test set were selected from the short videos, which increased the number of videos used to create the test set, increasing the test set’s diversity.

## 3. Dataset Evaluation/Experiments

We performed baseline experiments using video-based deep learning models to evaluate the difficulty in learning and predicting weather intensity using video clips. Given the absence of established methodologies for video-based weather recognition, we used the feature encoders of video-based deep learning methods and custom classification heads. Here, we outline the specifics of our training dataset, model architectures, and training methodology, and the validation outcomes are presented in Section 3.2.

### 3.1. Training Details

#### 3.1.1. Training Dataset

Table 2 provides a detailed breakdown (number of clips) of the training and testing dataset used for training models to predict the weather intensity. The number of clips used for training and validation is 5159 and 1583, respectively.

#### 3.1.2. Deep Learning Model Architecture

We trained numerous video-based deep learning models on the VARG dataset to predict weather intensity. The model architecture comprises two key components: the feature extractor or backbone and the classification head.

**Figure 5:**
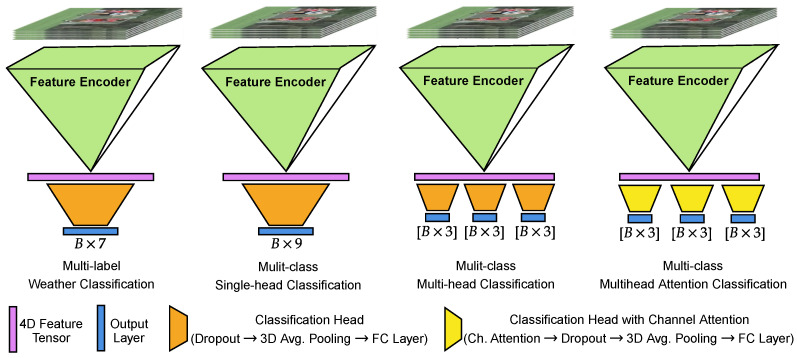
Model architecture with four different classification heads used for weather prediction. Here, “B” is the batch size.

- Feature encoders: For the feature extractor or backbone, we used the Temporal Shift Module (TSM) [20], Inflated 3D ConvNet (I3D) [21], Expanding 3D ConvNet (X3D) [22], VideoSWIN [23], and multi-scale vision transformer (MViTv2) [24], all pretrained on the Kinetic400 dataset [25]. We used the standard backbone architecture and the pretrained weights from PytorchVideo Library [26]. The output of the feature extractors is a 4D tensor with channel, time, height, and width dimensions, which is then used in the classification head for intensity classification. The dimension of feature vector is different for each backbone.
- Classification heads: Figure 5 illustrates the four custom classification heads used in this work: the multi-label head (MLH), the multi-class classification with single head (MCSH), the multi-class classification with multiple heads (MCMH), and the multi-class classification with multiple heads incorporating attention mechanisms (MCMHA):
  - MLH: It consists of a dropout layer, a 3D average pooling layer that pools the feature from time, height, and width dimensions, and a fully connected layer that outputs a vector of size seven followed by a sigmoid layer to generate predictions.
  - MCSH: Similar to MLH, MCSH consists of a dropout layer, a 3D average pooling layer, and a fully connected layer that outputs a vector of size nine. The output vector is reshaped into three rows, where each row uses a softmax layer to predict the intensity class for rain, fog, and snow weather.
  - MCMH: The MCMH classification head comprises three separate classification modules; each head consists of a dropout layer, a 3D average pooling layer, and a fully connected layer that outputs a vector of size three. The output from the three modules is passed through a softmax layer to individually predict the intensity of each weather condition.
  - MSMHA: Similar to MCMH, the MCMHA classification head also consists of three separate classification modules. Each module consists of a channel attention from the convolution block attention module [27], followed by a dropout layer, a 3D average pooling layer, a fully connected output layer which outputs a vector of size three, and a softmax layer to predict the intensity of each weather condition.

The models with MLH are trained with a multi-label annotation set, and a multi-class annotation set is used to train models with MCSH, MCMH, or MCMHA. We employed binary cross-entropy loss for architectures with multi-label heads and cross-entropy loss for architectures with multi-class heads.

#### 3.1.3. Training Procedure

In all experiments of intensity classification using videos, the deep learning models were trained using a stochastic gradient descent optimizer for 50 epochs with a learning rate of 0.001, which decays by a factor of 0.1 at epochs 30 and 40. Video augmentation was applied while training the networks, which includes RandAugment [28], normalization using the mean and standard deviation of the ImageNet data, random short side scaling followed by random crop to the desired size, and horizontal flipping with a probability of 0.5. During the training phase, video frames were randomly sampled and temporarily stacked. In the testing phase, frames were uniformly sampled, short-side-scaled to the desired size while maintaining the aspect ratio, center-cropped, and normalized using the mean and standard deviation of the ImageNet data. The dropout probability was set to 0.5.

#### 3.1.4. Training Platform

The training pipeline was implemented using the PyTorch library (version 1.13.1). All models were trained on an NVIDIA Tesla A100 graphics card, and the system comprised an Intel Xeon Gold CPU @ 2 GHz with 64 GB RAM. The batch size for training was adjusted based on the GPU memory and varied feature encoder.

### 3.2. Training Results

Table 3 provides the performance of various backbone architectures in conjunction with different classification heads across two key accuracy metrics: Hamming match and exact match. Hamming match measures the proportion of labels predicted correctly over all labels. In contrast, exact match measures the proportion of instances for which the set of predicted labels matches the ground truth.

Among the combinations tested, the multi-scale vision transformer (MViT-v2) backbone was the most accurate for both evaluation criteria. The MViT-v2 backbone with multi-label head (MLH) gave the best Hamming match (89.86%), while the MViT-v2 with multi-class multiple-head classification with attention module (MCMHA) pair obtained the best accuracy (68.22%) for exact match criteria. These results suggest that when used with the multi-class single-head classification, the MViT-v2 backbone is particularly effective for accurately predicting weather intensity across different conditions.

On the other hand, the Temporal Shift Module (TSM) backbone combined with the multi-label head (MLH) showed comparatively lower performance, achieving an accuracy of 87.93% in Hamming match and 44.79% in exact match scenarios. This combination appears less effective than others, indicating potential limitations in capturing the complexities of weather intensity prediction by the 2D CNN modules with temporal shifts compared to other 3D-CNN approaches like X3D and I3D model architectures.

The best classification results were obtained using transformer architectures, which outperformed the other backbones. Concerning the classification head, the evaluation based on exact match criteria reveals that multi-class classification outperformed multi-label classification for all the backbones; furthermore, multi-class classification using a single head was better than other tested classification heads by >5%, while for Hamming match criteria, multi-label classification was best. Since precise prediction for each weather condition is imperative, exact matching criteria offer a more stringent evaluation standard, consequently yielding more favorable results.

**Figure 6:**
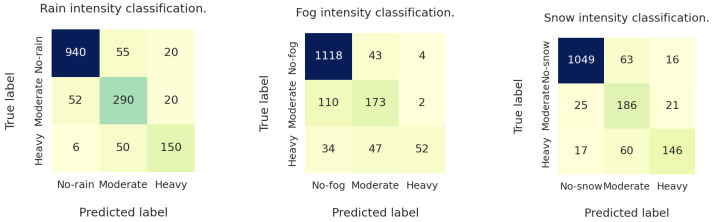
Confusion matrices of multi-class weather intensity classification using MViTv2 backbone and multi-class multiple-head classification with attention module (MCMHA) that provided the best exact match accuracy on the test dataset. Darker color correspond to larger value.

Figure 6 presents the confusion matrix of intensity prediction for each weather label, corresponding to the best result (MViT-v2 backbone with MCSH) obtained on the test dataset. The matrix shows that the biggest number of misclassifications occurred for the cases of moderate and heavy fog intensities.

## 4. Discussion

In this study, we present VARG, a novel multi-label dataset designed for weather intensity prediction, and perform an extensive evaluation of weather intensity prediction using video clips. Accurate information regarding weather and intensity is useful for making critical decisions and ensuring safe autonomous driving under adverse weather conditions, such as establishing sensor reliability and determining confidence thresholds for object detection.

The experimentation aims to validate dataset labels and investigate the concept of weather intensity prediction using videos. Specifically, we investigate the impact of various state-of-the-art video recognition backbone architectures (including TSM, I3D, X3D, MViT, and Swin models) and the approach of weather intensity classification as a multi-label or multi-class classification problem.

As depicted in Table 3, the findings indicate that using a transformer backbone leads to the highest accuracy for multi-label and multi-class weather intensity prediction. The better performance of transformer-based architectures is expected, as transformers outperform convolutional neural networks in capturing visual complexity due to the employed self-attention mechanism.

Notably, weather intensity prediction, when approached as a multi-class classification, demonstrates the best results on exact match metrics, even with a single classification head. The lower performance of multi-label classification could be attributed to the possibility of the co-occurrence of moderate and heavy weather intensity labels for a single weather type, which can be avoided in multi-class classification problems. This is evident in Table 3, where the results for every multi-classification method outperform the multi-label accuracy for the exact match metric.

**Figure 7:**
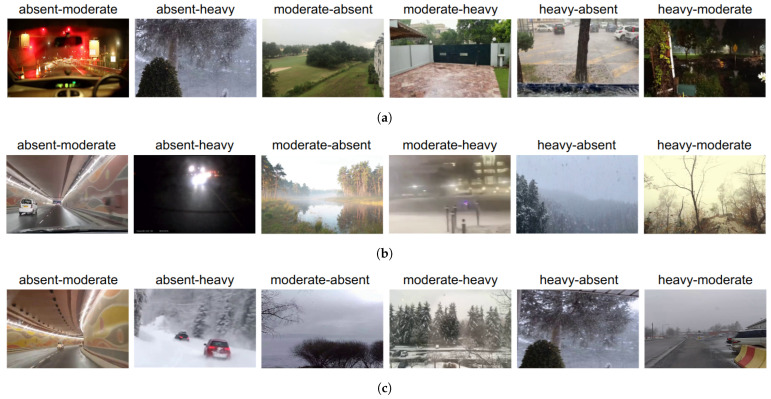
Examples of wrong classifications of weather intensity for (**a**) rain, (**b**) fog, and (**c**) snow weather. The ground truth and predicted labels for each image are given on top of the images in the format “true-predicted”. (**a**) Rain intensity misclassification. (**b**) Fog intensity misclassification. (**c**) Snow intensity misclassification.

Analyzing the misclassified results depicted in Figure 7, we observed inaccuracies in rain and snow weather intensity predictions when the video contains other adverse weathers, snow and rain, respectively. However, limiting the analysis to only clear weather significantly reduces inaccuracies between clear and heavy weather intensity labels for snow and rain. Similarly, misclassification between fog and clear weather often arises due to the presence of rain and snow (even on-ground snow) in the videos. Additional factors contributing to inaccurate weather intensity predictions include nighttime videos with low illumination and instances where weather information is visible only in certain parts of the video, indicating the dataset’s diversity. These challenges in weather intensity prediction present opportunities for enhancing results by improving the distinction between rain and snow weather and distinguishing foggy weather from clear weather images or images with fewer features (for example, images with lots of on-ground snow).

In conclusion, this study presents a unique video dataset for weather intensity prediction, covering a wide spectrum of scenarios. We provide an evaluation study that demonstrates the value of weather intensity prediction and discuss the challenges encountered in multi-weather intensity prediction, highlighting common causes of incorrect predictions. We are certain that this dataset will be a valuable asset for the computer vision community.

### Limitation

While the dataset presented in this work fills a gap in weather detection research using camera sensors and provides a challenging dataset for video-based classification research, it is essential to acknowledge one major limitation of the current data creation approach: manual labeling. Manual labeling incorporates the personal bias of the annotator and restricts the use of this approach for dataset expansion. Manual labeling was necessary due to the lack of a more accurate method for estimating weather intensity. However, based on the accuracy of the prediction for single weather intensity prediction, the trained models could serve as preannotation tools to expedite data labeling for existing autonomous driving datasets in the future.

## 5. Conclusions

In conclusion, this paper introduced the VARG dataset, a unique dataset for predicting weather intensity with potential weather monitoring applications for agricultural scenarios, drone technology, and autonomous driving scenarios. Additionally, we conducted a comprehensive evaluation of weather intensity prediction using video clips. Through experiments, we demonstrated the effectiveness of video clips for predicting weather intensity. The best-achieved prediction accuracy was 89.86% for Hamming match criteria using the MViTv2 model with a multi-label classification head. In the case of an exact match metric, the best accuracy achieved was 68.22% using the MViT-v2 feature encoder with multi-class multiple-head classification with channel attention module (MCMHA). However, we also identified specific challenges in accurately predicting moderate and heavy intensities and distinguishing fog from other weather conditions. It is important to note that the manual labeling approach used in creating the VARG dataset introduces some limitations due to the personal bias of the annotator. Nonetheless, the trained models can serve as valuable pre-annotation tools to expedite data labeling in the future, potentially mitigating this limitation. Furthermore, the dataset’s availability to the research community can facilitate the development and evaluation of novel algorithms and techniques in this important domain, ultimately contributing to safer and more reliable autonomous driving systems in adverse weather conditions.

## 6. Future Work

In future work, we aim to expand the VARG dataset by adding more video clips for weather conditions like snow, fog, and rain. Additionally, we plan to introduce new weather conditions, such as hail and sandstorms, to address adverse scenarios encountered in real-world applications.

## Figures and Tables

**Table 1:** The distribution of clips in the VARG dataset for each weather label. The blue and red rectangles show the combination of weather labels that co-occur in the clips. The number of clips with rain and fog labels is 49 and with snow and fog labels is 1495.

Weather	Moderate	High	Total
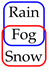	1355	839	2194
	1532	571	2703
	1423	912	2335
Clear			1654

**Table 2:** Detailed decomposition (number of clips) of the training dataset for weather intensity classification.

	Train		Test	
	Moderate	Heavy	Moderate	Heavy
Rain	993	633	362	206
Fog	1247	438	286	133
Snow	1191	689	232	223
Clear	1218		436	

**Table 3:** Accuracy (%) for Hamming match and exact match of weather intensity prediction for all the trained models on the test dataset for various combinations of feature encoders (Temporal Shift Module (TSM), Inflated 3D ConvNet (I3D), Expanding 3D ConvNet (X3D), VideoSWIN, and multi-scale vision transformer (MViTv2)) and classification heads (multi-label head (MLH), multi-class single head (MCSH), multi-class multiple heads (MCMH), and multi-class multiple heads with attention module (MCMHA)). Bold number shows the best accuracy for each classification head with different feature encoders.

	Hamming Match				Exact Match			
	MLH	MCSH	MCMH	MCMHA	MLH	MCSH	MCMH	MCMHA
TSM	87.93	82.06	82.54	82.48	44.79	59.63	59.95	60.33
I3D	88.79	83.98	83.18	83.15	48.39	63.04	61.66	60.33
X3D	88.07	84.50	83.87	83.09	42.07	63.55	62.48	60.64
MViT	**89.86**	**85.55**	**86.10**	**86.42**	55.21	**66.71**	**67.59**	**68.22**
Swin	89.81	83.98	83.55	83.68	**57.11**	63.11	62.35	62.35

**Figure A1:**
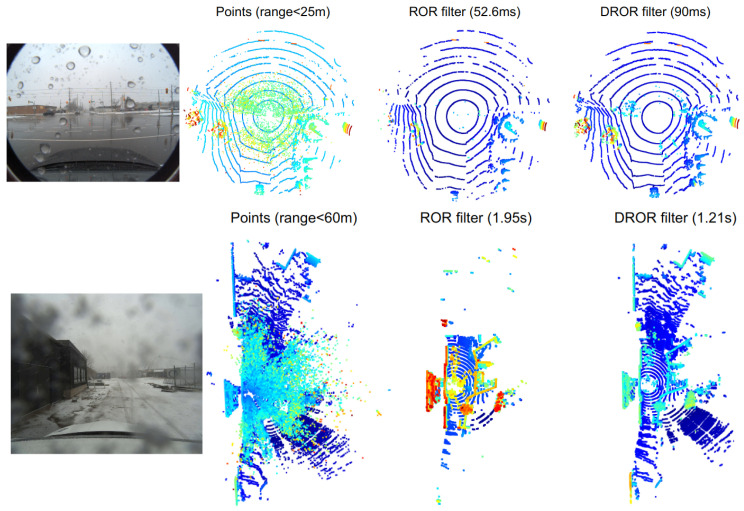
Lidar scans and efficiency of ROR and DROR filters in moderate (**top row**) and heavy snow (**bottom row**). Source: sequence “2018_03_07” of the CADC dataset for **top row**, and sequence “2021_01_26” of the Boreas dataset for **bottom row**.

**Figure A2:**
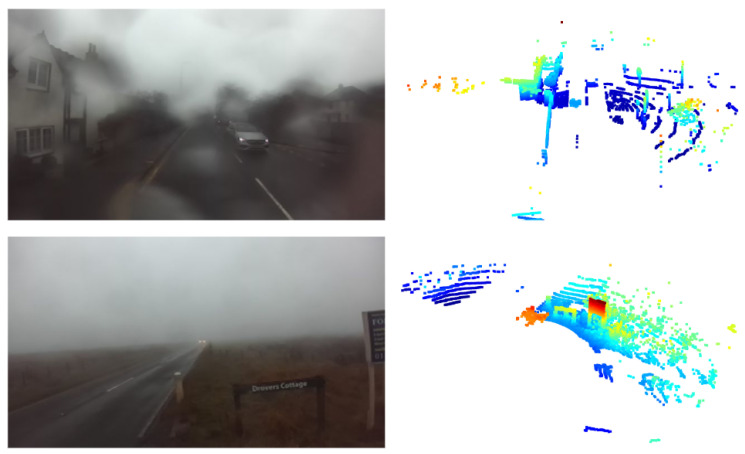
Lidar and camera in moderate (**top row**) and heavy (**bottom row**) fog. Source: sequence “fog_6_0”, and sequence “fog_8_0” from Radiate dataset for **top row** and **bottom row**. The maximum point range for lidar scans in moderate and heavy fog is 50 and 18 m, respectively, in this particular case.

**Figure A3:**
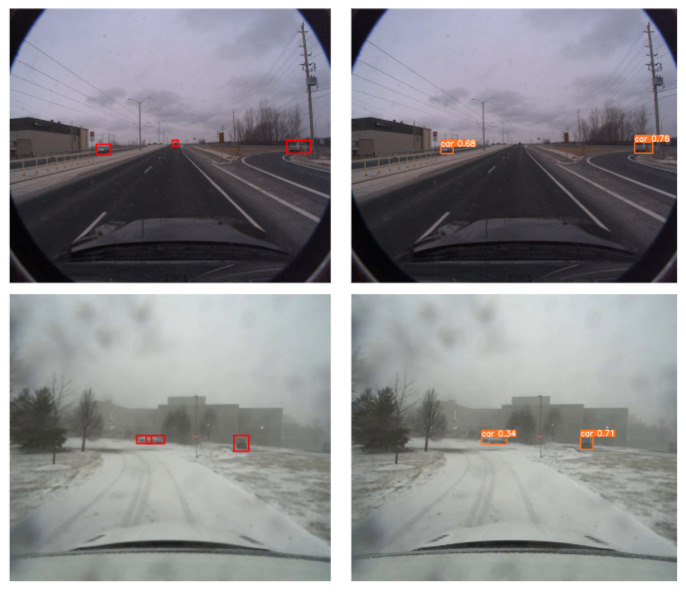
Result of object detection using Yolov5 in “moderate” and “heavy” snowing shown in 1st and 2nd row, respectively. Red boxes indicate the ground truth and orange (car) boxes shows the prediction using Yolov5 model.

**Figure A4:**
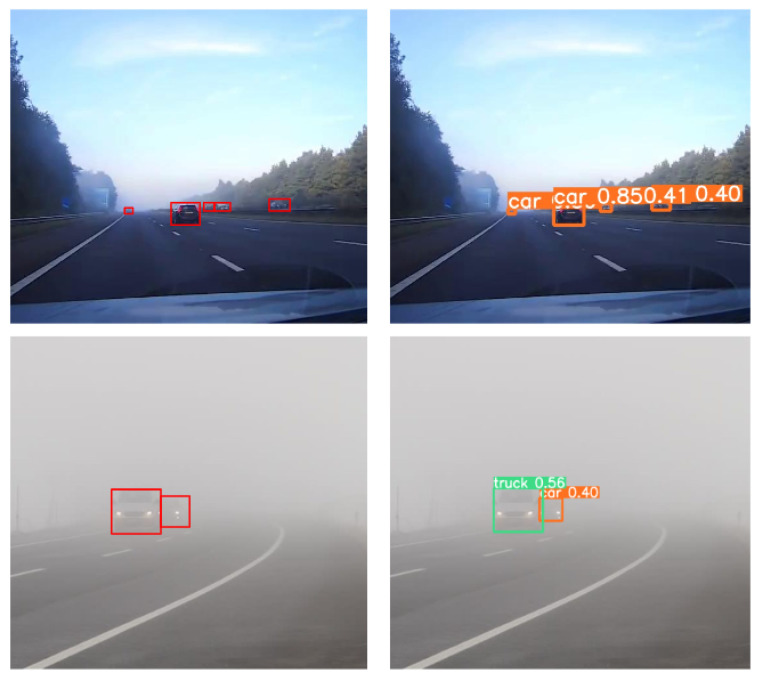
Result of object detection using Yolov5 in “moderate” and “heavy” fog shown in 1st and 2nd row, respectively. Red boxes indicate the ground truth and green (truck) and orange (car) boxes shows the prediction using Yolov5 model.

## Data Availability

The data created and used in this study along with the code are available at https://github.com/hgupta01/Weather_Intensity_Recognition.git (accessed on 6 October 2024).

## Appendix A. Vision Sensors in Adverse Weather

### Appendix A.1. Lidar in Adverse Weather

The impact of adverse weather on lidar sensors is contingent upon the specific weather conditions and their corresponding intensity levels. In scenarios involving rain and snow, the lidar data often contain noisy points due to reflection from raindrops and snowflakes, the amount of which is affected by the severity of the weather intensity and needs to be removed for downstream tasks like scan registration, object detection, localization, etc.

For moderate rain or snow, the density of noise points is relatively low, primarily congregating near the sensor. Standard point cloud denoising algorithms like radius outlier removal (ROR) can effectively eliminate a substantial portion of this noise. However, in cases of heavy intensity, the noisy points exhibit a high density, and they can extend far from the sensor, necessitating more sophisticated filtering techniques like dynamic radius outlier removal (DROR). To illustrate this phenomenon, Figure A1 visually depicts the influence of snow intensity on lidar scan noise. Moreover, the figure underscores how the choice of noise filtration technique, determined by the specific snow intensity, impacts both processing time and noise reduction efficacy.

Unlike the sparse noise encountered in rain and snow conditions, dense fog or smoke act as a wall for lidar sensors operating in fog or smoke. Automotive lidars generally function within a range of less than 200 m, while the visibility range for low–moderate fog is >100 m and for dense fog is <100 m. The preprocessing of point cloud data necessitates a distinct approach under such conditions. Removing points with a range surpassing a certain threshold is easier in low to moderate fog intensity. However, with dense or heavy fog, a substantial portion of the point cloud data is impractical for use, as illustrated in Figure A2. For point range measurement, we used the point cloud provided in the dataset without any filtering. This figure visually contrasts the filtered point cloud in situations of moderate and heavy fog, underlining the challenges of point cloud preprocessing in dense fog or smoke conditions.

### Appendix A.2. Camera Sensor in Adverse Weather

The preceding figures show the impact of adverse weather on camera sensor images. Alongside the discernible patterns of weather-induced noise, supplementary noise effects such as haziness, water droplets, or frost accumulation on camera lenses also manifest. The literature offers an array of algorithms tailored for denoising adverse weather images. A distinct investigation is required to comprehensively examine how downstream computer vision tasks perform on denoised images, including object detection and image segmentation.

We focused on assessing the confidence scores for object detection using the pre-existing Yolov5-l model to simplify the utility of weather intensity information for camera sensors. Figure A3 and Figure A4 illustrate that object detection, even in severe cases, might not be the problem if object feature are present. Instead, the problem lies with the confidence score associated with these detections. In the case of heavy snow (as depicted in the bottom image of Figure A3), the prediction score for a nearby vehicle is only 0.3. Similarly, in instances of heavy fog (illustrated in the bottom image of Figure A4), both nearby vehicles were successfully detected, but the associated confidence levels barely surpassed 0.55. These concise experiments underscore the tangible impact of weather intensity, even on camera sensors, reinforcing the significance of weather intensity information.
